# GnRH receptor mediates lipid storage in female adipocytes via AMPK pathway

**DOI:** 10.7150/ijms.74335

**Published:** 2022-08-15

**Authors:** Xiaoyong Li, Xueyan Zhang, Zhaojun Shen, Zhengyun Chen, Hanzhi Wang, Xinmei Zhang

**Affiliations:** 1 Department of Gynecology, Women's Hospital School of Medicine Zhejiang University, Hangzhou 310006, Zhejiang, People's Republic of China.; 2 Department of Obstetrics and Gynecology, The Fourth Affiliated Hospital, School of Medicine Zhejiang University, Yiwu, 322000, Zhejiang, People's Republic of China.; 3 Central laboratory, Women's Hospital School of Medicine Zhejiang University, Hangzhou 310006, Zhejiang, People's Republic of China.

**Keywords:** Gonadotropin-releasing hormone, gonadotropin-releasing hormone receptor, adipocytes, AMP-activated protein kinases, obesity, women

## Abstract

**Objective:** Due to high levels of serum gonadotropin-releasing hormone (GnRH), perimenopausal or menopausal women, girls with central precocious puberty, women of polycystic ovary syndrome, and females receiving long-term GnRH agonist (GnRHa) treatment are at substantially higher risk of developing obesity. However, it remains poorly understood how GnRH affects body weight. Here, we explored whether the gonadotropin-releasing hormone receptor (GnRHR) was expressed in adipocytes and how GnRHR mediated lipid accumulation and the development of obesity.

**Methods:** The samples were from 18 patients with benign tumors operated between 01/2018 and 06/2018 at the Women's Hospital School of Medicine Zhejiang University. Immunofluorescence, Western Blotting, and RT-PCR were used to detect whether the GnRH receptor was expressed in the specimens and human preadipocytes-subcutaneous (HPA-s). The GnRH receptor agonist diphereline with different concentrations was used to stimulate the HPA-s cells for 24, 48, and CCK-8 was used to detect cell proliferation. Oil red-O staining was used to detect lipid droplets in mature adipocytes. The phosphorylation of AMPK-Ser485/Thr172 was detected by Western Blotting.

**Results:** GnRH receptor was expressed in all 18 human subcutaneous adipose tissue specimens. Cultured HPA-s expressed the GnRH receptor, and the expression increased during the process of cell maturation. The GnRH receptor agonist diphereline can stimulate the proliferation of HPA-s cells, which can advance the earliest occurrence of lipid droplets in HPA-s cells and the occurrence of lipid droplets in 50% cells by 1-2 days. Diphereline can stimulate the increase in the number of lipid droplets in mature adipocytes. The phosphorylation level of AMPK-Ser485/Thr172 in mature adipocytes was decreased by diphereline.

**Conclusion:** The GnRH receptor was expressed in adipocytes. As adipocytes mature, GnRH receptor expression gradually increased. GnRHa stimulates the proliferation of HPA-s, promotes adipocyte maturation, increases the formation of lipid droplets in mature adipocytes, and inhibits the activation of the AMPK pathway in adipocytes. Our findings may elucidate the mechanism of obesity in these female populations and provide some evidence on how GnRH contributes to obesity. Additionally, these results provide theoretical support for further research on the mechanisms of obesity, thus enhancing our understanding of the functional diversity of GnRH and establishing a new theoretical basis for the impact of GnRH on metabolism.

## Introduction

With the development of the social economy, obesity has become a global epidemic trend 1. It was reported that about two billion people worldwide suffered from obesity 2. Obesity and its related diseases, such as diabetes, cardiovascular and cerebrovascular diseases, thrombosis, gallstones, sleep-respiratory disorder syndrome, and cancer, have become a severe public health problem 3-7. The fat mass can expand by increasing the average fat cell volume and the number of adipocytes. Adipocytes can synthesize and store fat, but cannot proliferate. Preadipocytes can proliferate and differentiate into adipocytes, but cannot synthesize and store fat themselves. The total number of adipocytes is different between lean and obese individuals, is established during childhood and adolescence, and stays constant during adulthood. Increased fat storage in mature adipocytes, resulting in enlarged fat cells, is thought to be the most important mechanism of obesity 8. Although many approaches have been successfully applied for weight control, many patients are prone to rebound, especially perimenopausal and menopausal women.

Gonadotropin-releasing hormone (GnRH) is a decapeptide hormone secreted by the hypothalamus. Its sequence is highly conserved in the mammals, and its main physiological function is to promote the synthesis and secretion of luteinizing hormone (LH) and follicle-stimulating hormone (FSH) by activating GnRH receptor in pituitary cells, which in turn act on reproductive organs like ovaries or testes to regulate gametogenesis and sex hormone secretion, finally forming hypothalamus-adenopituitary-gonad axis 9. GnRH receptor is mainly expressed in pituitary cells and some extra pituitary tissues such as ovaries, myometrium, endometrium, breast, prostate, placenta, and sex hormone-sensitive tumors, such as ovarian cancer, endometrial cancer, breast cancer, and prostate cancer 10-14. Compared with women of normal childbearing age, the incidence of obesity is significantly higher in perimenopausal or menopausal women and patients with central precocious puberty, polycystic ovary syndrome, and long-term treatment of GnRH agonist (GnRHa) 15-18. Increased GnRH is found in such patients. GnRHa can bind and activate GnRH receptors, but continuous administration or long-acting GnRH agonists overriding the physiologic pulsatile GnRH secretion leads to desensitization of pituitary GnRH receptor and the corresponding inhibition of gonadotrophin and gonadal hormones secretion, so GnRHa is often used to treat estrogen-dependent diseases such as pelvic endometriosis, precocious puberty, and breast cancer 19-21. Patients undergoing a long-term treatment of GnRHa (i.e., over 6 months) may have decreased muscle content, increased body fat rate, and trunk/limb fat ratio 22, suggesting that high GnRH level might be positively associated with obesity. However, it is still unclear how GnRH affects body weight.

The AMPK signaling pathway has a key role in regulating cellular energy homeostasis. AMP-activated protein kinases (AMPK) belong to the AMP-dependent protein kinase family, the activation of the AMPK signaling pathway by phosphorylation has been reported to promote lipolysis and inhibit fat synthesis in adipocytes 23, 24. However, whether the AMPK pathway affects obesity through GnRH remains elusive.

Consequently, in the current study, we identified the expression of GnRH receptor in human adipose tissues, investigated the expression of GnRH receptor during the induction of adipocyte maturation, explored the role of GnRH receptor in the process of adipocyte maturation, and elucidated the effect of GnRH receptor on the production of lipid droplet during the process of adipocyte maturation to address the above-listed issues. Our results provided evidence that activation of the GnRH receptor promoted the development of obesity. These findings further explained the pathological processes in many diseases, including the high incidence of obesity in perimenopausal or menopausal women and patients with central precocious puberty, polycystic ovary syndrome, and long-term treatment of GnRHa.

## Materials and methods

### Samples Collection

The samples were taken from 18 women with benign tumors who were operated by laparotomy at the Women's Hospital School of Medicine Zhejiang University between January 2018 and June 2018. The patients were 45.0±9.9 years of age and had a body mass index (BMI) of 24.3±3.4 kg/m^2^. Only patients with benign diseases (such as uterine fibroids, ovarian cysts, adenomyosis, and endometrial hyperplasia) were selected. As gynecological cancers often overexpress the GnRH receptor, patients with ovarian cancer, endometrial cancer, or breast cancer were excluded.

This study was approved by Ethics Committee of Women's Hospital Affiliated to Zhejiang University School of Medicine (#20160102), and all participants provided written informed consent. The protocol was approved by the Ethics Committee of Animal Ethics Committee, Zhejiang University Medical College (ZJU20170365). This work has been carried out in accordance with the Declaration of Helsinki (2000) of the World Medical Associatio.

A sample of subcutaneous fat tissue about 2×1×1 cm in size was taken under the abdominal incision before the subcutaneous fat was sutured at the end of the operation. Specimens were immediately divided into three parts. Two parts were put into a freezing tube filled with liquid nitrogen for 10 min and then transferred at -80 °C for storage. Another part was put into 10% formalin and fixed for 12-24 h.

With the approval of the Animal Ethics Committee of Zhejiang University (ZJU20170365) three female adult SD rats weighing about 250g were taken from the Laboratory Animal Center of Zhejiang University in a specific pathogen-free environment. They were sacrificed by spinal dislocation, and the pituitary gland was taken. Specimens were treated as described above.

### Immunohistochemical detection of GnRH receptor in adipose tissue

Formalin-fixed human adipose specimens were routinely embedded in paraffin, sectioned, and mounted on slides. Antigen repair was conducted through the high-temperature and high-pressure methods. Endogenous peroxidase was blocked using 3% H_2_O_2_. The sections were reacted with rabbit polyclonal antibody GnRHR (ab202848; 1:250; Abcam, Cambridge, United Kingdom) at 37 °C for 1 h. PBS was used as blank control, and rat pituitary sections were used as a positive control. The goat anti-rabbit IgG (H+L) secondary antibody (#31210; 1:5000; Thermo Fisher Scientific, Waltham, MA, USA) was incubated at 37 °C for 30 min and washed with PBS. The proteins were revealed using the DAB color development solution for 5 minutes. The nuclei were stained with hematoxylin for 1 min. The sections were dehydrated with 95% and 100% ethanol, transparent in xylene, and sealed with neutral gum. The observation was performed under a DM3000 microscope (Leica Microsystems, Wetzlar, Germany).

Rat pituitary tissue was used as positive control, and adipose tissue section without primary antibody was used as negative control.

### Western blot analysis of GnRH receptor in adipose tissue

Adipose tissue specimens and rat pituitary specimens were placed in a mortar and ground. The total protein of adipose tissue specimens was extracted using the Minute Total Protein Extraction Kit for Adipose Tissues/Cultured Adipocytes (AT-022, Invent Biotechnologies, Inc., Plymouth, MN, USA), and total pituitary protein was extracted using the T-PER Tissue Protein Extraction Reagent (78510, Thermo Fisher Scientific, Waltham, MA, USA). For cultured adipocytes, the total protein was extracted using the Minute Total Protein Extraction Kit for Adipose Tissues/Cultured Adipocytes (AT-022, Invent Biotechnologies, Inc., Plymouth, MN, USA). All operations were performed according to the manufacturers' instructions. The protein concentrations were determined using a BCA protein concentration determination kit (Beyotime Institute of Biotechnology, Haimen, China). The ECL DualVue WB Marker (RPN810, GE) was used. The proteins (50 µg) were resolved by polyacrylamide gel electrophoresis and transferred to PVDF membranes (IPVH00010; Millipore Corp., Billerica, MA, USA). The membranes were probed with antibodies against GnRHR (ab202848; 1:500, Abcam, Cambridge, United Kingdom), GAPDH (ab181602; 1:7000, Abcam, Cambridge, United Kingdom), AMPK (ab3759; 1:500, Abcam, Cambridge, United Kingdom), p-AMPKα (Thr172) (40H9) (#2535; 1:500, Cell Signaling Technology, Inc., Danvers, MA, USA), and p-AMPKα (Ser485) (45F5) (#2537; 1:500, Cell Signaling Technology, Inc., Danvers, MA, USA) at 4 °C overnight, followed by the secondary antibody goat anti-rabbit IgG (H+L) secondary antibody (#31210; 1:5000, Thermo Fisher Scientific, Waltham, MA, USA) for 1 h at room temperature. The proteins were revealed using the SuperSignal West Dura Extended Duration Substrate (#34075; Thermo Fisher Scientific, Waltham, MA, USA).

Protein extracted from rat pituitary specimens was selected as positive control. The negative control is the loading buffer.

### Real-Time PCR of GnRH receptor in adipose tissue

Adipose tissue specimens were ground in a mortar using a small amount of liquid nitrogen. Total RNA was extracted using TRIzol^®^ Plus RNA Purification Kit (#12183-555; Invitrogen Inc., Carlsbad, CA, USA) and added with RNase-Free DNase Set (#79254; Qiagen, Venlo, The Netherlands). cDNA was synthesized using the SuperScript™ III First-Strand Synthesis SuperMix for qRT-PCR (#11752-050; Invitrogen Inc., Carlsbad, CA, USA). All RT-PCR analyses were performed using the Power SYBR® Green PCR Master Mix (#4367659; Applied Biosystems, Foster City, CA, USA), according to the manufacturer's instructions). The primers were: GnRH receptor forward primer 5'-CCT TAG CCA ACC TGT TGG AGA CT-3' and reverse primer 5'-GGC TGG GGC ATA CAT GGA GAA AA-3', and GAPDH forward primer 5'-CCA TGA CAA CTT TGG TAT CGT GGA A-3' and reverse primer 5'-GGC CAT CAC GCC ACA GTT TC-3'. The reaction mixture was made of 8.0 µL of water, 10.0 µL of SYBR Green Master Mix, 0.5 µL of each primer, and 1.0 µL of cDNA. The reaction conditions were: 1) 95 °C for 1 min; 2) 40 cycles of 95 °C for 15 s, 60 °C for 25 s. The melting curve was obtained at 55-95 °C. The GnRH receptor-encoded gene product was amplified by RT-PCR, separated and identified by 2% agarose gel electrophoresis, and sent to Sangon Biotech (Shanghai, China) for sequencing (Supplementary material).

### Culture, induction, and maintenance of human preadipocytes-subcutaneous

Human preadipocytes-subcutaneous (HPA-s) were cultured in a specific medium (#7220; Sciencell Research Laboratories, Carlsbad, CA, USA), strictly adhering to the manufacturer's instructions. They were induced into mature adipocytes using the preadipocyte differentiation medium (#7221; Sciencell Research Laboratories, Carlsbad, CA, USA) and maintained in adipocyte medium (#7201; Sciencell Research Laboratories, Carlsbad, CA, USA).

### GnRH receptor expression in HPA-s cells and adipocytes

A 6-well plate was inoculated with 3×10^5^ cells/2ml/well and incubated overnight. The HPA-s were induced with the induction medium for 3, 6, 9, 12, 15, and 18 days. Non-induced HPA-s were used as control. PBS was used for rinsing for 5 mins, 4% fresh paraformaldehyde was used for fixation at 4 °C for 30 mins, PBS was used for rinsing for 5 mins three times, and the cells were incubated for 30 mins in 10% Donkey serum. The GnRH receptor was detected using rabbit polyclonal GnRHR Antibody (19950-1-AP; 1:250; Proteintech Group Inc., Chicago, IL, USA) and incubated overnight at 4 °C. Alexa Fluor 488 Donkey Anti-Rabbit IgG (715-545-152; 1:200; Jackson ImmunoResearch, West Grove, PA, USA) was incubated at room temperature for 1 h. The cells were incubated with DAPI at room temperature for 2 mins. The FluorSave Reagent (#345789; Merck Frosst, Montreal, Canada) was added to seal the slides, which were then photographed by an LSM 710 laser scanning confocal microscope at 630× (Carl Zeiss GmbH, Oberkochen, Germany). A negative control was used also, that is, no primary antibody was added during the cytofluorescence immunoassay and the other operations were exactly the same.

HPA-s cells were induced by the same method for 3, 6, 9, 12, 15, and 18 days, washed, digested, centrifuged, collected, and total protein and mRNA were extracted. Non-induced HPA-s were used as control. Western blot and RT-PCR were used to detect the expression of the GnRH receptor (as above for tissue specimens), and the differences were compared.

### Effect of GnRH receptor activation on the proliferation of HPA-s

HPA-s cells were inoculated into 96-well plates (3×10^4^ cells/200 µl/well) and were cultured overnight. Different concentrations of GnRHa diphereline (10^-11^, 10^-10^, 10^-9^, 10^-8^, 10^-7^, 10^-6^, and 10^-5^ mol/L) were added. All experiments were performed in triplicates. The cells were incubated for 24 and 48 h, and the cell proliferation rate was detected by the CCK-8 method (WST-8, Abcam, Cambridge, United Kingdom) according to the manufacturer's instructions. The proliferation rate was calculated using the following formula: (experimental group OD value-blank control OD value)/(negative control OD value-blank control OD value) × 100%.

### Effect of GnRH receptor activation on the accumulation of lipid droplet

HPA-s cells were induced as above and different concentrations of diphereline were added into the culture medium. The cells were stained with oil red-O after 18 days of induction. The fixed cells were rinsed with isopropanol 60%, stained for 15 min with freshly prepared Oil Red O solution, rinsed with isopropanol 60%, stained with hematoxylin, and then rinsed in water. The lipid droplets were dyed red, and the formation of lipid droplets was observed under an optical microscope (400×). The lipid droplets were dyed red and observed under a microscope. Quantitative analysis of Oil Red O Staining intensity (Integrated Optical Density/area) was performed using Image-Pro Plus 6.0 software (Media Cybernetics, USA).

### Effect of GnRH receptor activation on AMPK pathway

The HPA-s were inoculated into 6-well plates, induced with the induction medium for 18 days, and then changed into adipocyte medium with different concentrations of GnRHa diphereline (10^-11^, 10^-10^, 10^-9^, 10^-8^, 10^-7^, 10^-6^, and 10^-5^ mol/L) for 5 days, the cells untreated with diphereline were used as control. The cells were washed, digested, centrifuged, collected, and total protein was extracted. AMPK, p-AMPKα (Thr172) and p-AMPKα (Ser485) were tested by Western blot. Similarly, the levels of AMPK in adipocytes of different maturity were detected.

### Statistical analysis

SPSS 20.0 (IBM, Armonk, NY, UDS) was used for statistical analysis. Analysis of variance for repeated measurements and the post hoc LSD test were used to examine for differences. Two-sided P-values <0.05 were considered statistically significant.

## Results

### GnRHR was highly expressed in human adipose tissues

**Figure 1:**
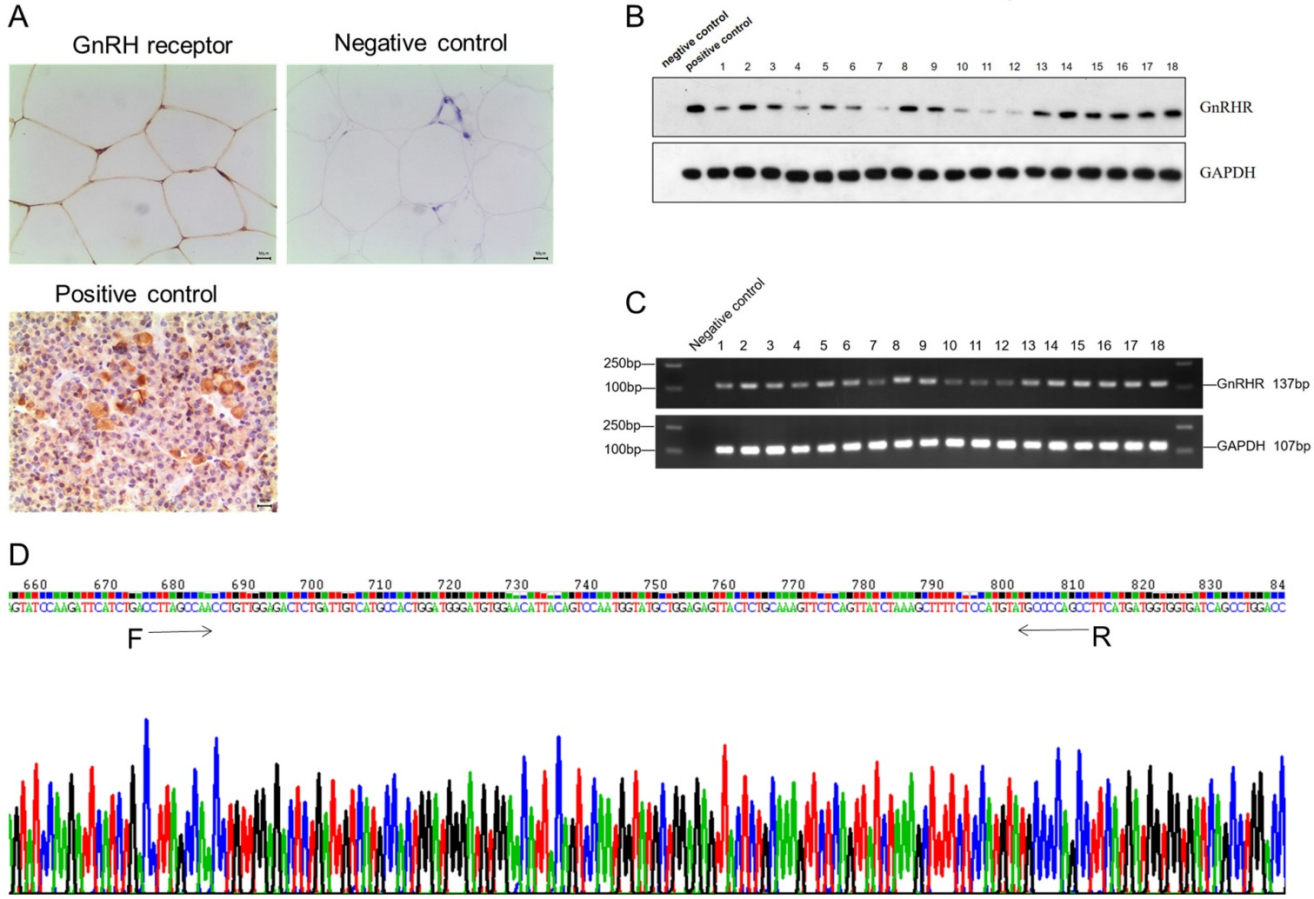
** GnRH receptor were highly expressed in human adipose tissues. (A)** The expression of the GnRH receptor was detected by immunohistochemistry. **(B)** Western blot showed the high protein level of the GnRH receptor. **(C)** High mRNA expression of the GnRH receptor was detected by RT-PCR analysis. **(D)** Sequencing results of RT-PCR products of GnRH receptor gene fragments, and it was confirmed to be consistent with the GnRH receptor gene by gene alignment.

To identify the expression of GnRH receptor in human adipose tissues, we collected normal subcutaneous fat tissue from 18 patients. Immunohistochemistry results showed GnRH receptor was highly expressed on the membrane of adipocytes in human adipose tissue (Figure 1A), which was further validated by PCR and Western Blotting (Figure 1B and 1C). Furthermore, all RT-PCR products of GnRHR gene fragments were sequenced and confirmed after being aligned with its sequence on Pubmed (Figure 1D). All these data suggested that GnRH receptor are highly expressed in human adipose tissues.

### The expression of GnRHR was increased during the adipocyte maturation

**Figure 2:**
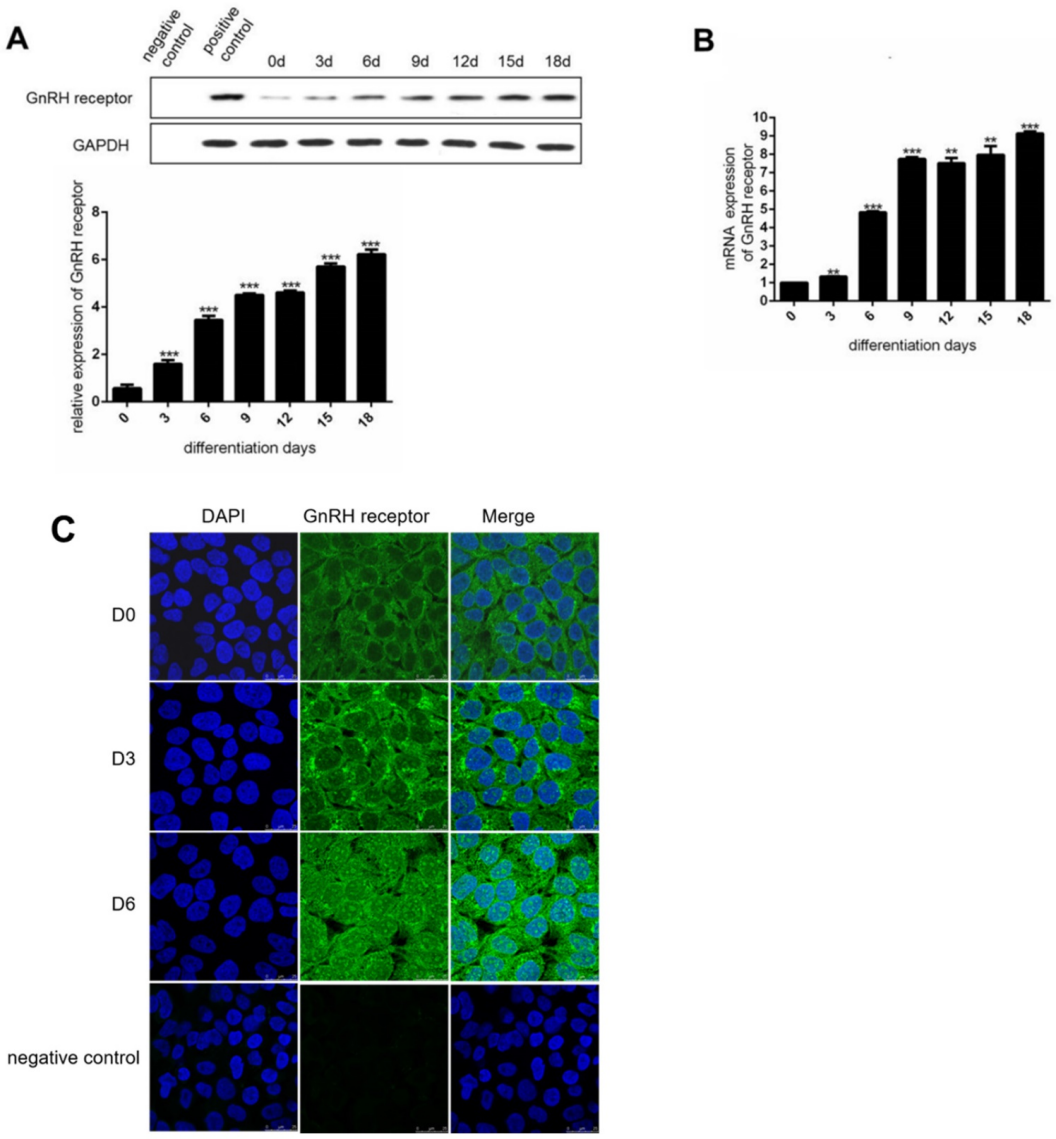
** The expression of GnRH receptor is increased during the induction of adipocytes. (A)** Western Blot analysis for the protein expression of the GnRH receptor in HPA-s during their maturation into adipocytes. **(B)** RT-PCT analysis for the mRNA expression of the GnRH receptor in HPA-s during their maturation into adipocytes. **(C)** Immunofluorescence analysis of the GnRH receptor expression after 6 days of induction (***P<0.001, **P<0.01).

To investigate the expression of GnRHR during the induction of adipocyte maturation, we used human preadipocytes-subcutaneous (HPA-s) for the induction of mature adipocytes. During the induction process, we found that the expression of GnRHR was dramatically increased (Figure 2A and 2B). Specifically, the expression of GnRH receptor was increasingly stronger in the cell membrane and cytoplasm by immunofluorescence staining (Figure 2C). Interestingly, the increased expression of GnRH receptor in the nucleus during the induction was also observed. These data demonstrated that GnRH receptor could be gradually induced to express during the process of adipocyte maturation.

### Stimulating GnRHR can influence the proliferation of HPA-s

**Figure 3:**
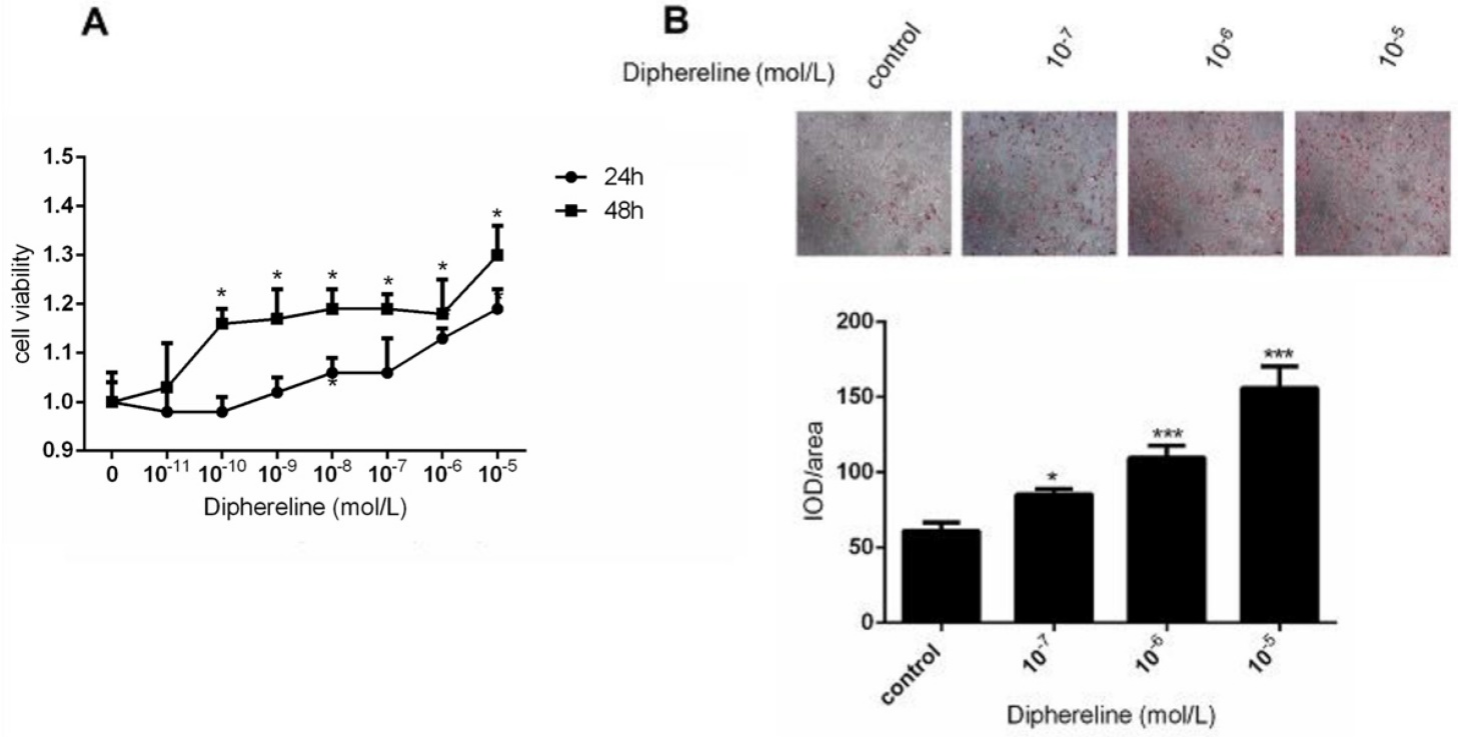
** Effect of GnRHa on the proliferation and differentiation of human adipocytes.** Human preadipocytes-subcutaneous (HPA-s) were induced into mature adipocytes and treated using GnRHa. **(A)** Cell viability using different concentrations of diphereline and different treatment durations. The cells have not been induced, and their proliferation was tested after being stimulated by diphereline. **(B)** Oil Red O staining of the adipocytes. The precursor cells were stimulated by diphereline during the differentiation process. After they differentiated into mature adipocytes, they were stained with oil red to detect the storage of lipid droplets (*P<0.05; ***P<0.001).

To explore the role of GnRH receptor on the number of adipocytes, we treated HPA-s with different concentrations of GnRH receptor agonist diphereline that can activate the GnRH receptor. In the first two days of diphereline treatment, we found that diphereline could stimulate HPA-s proliferation in a time and dose-dependent manner (Figure 3A), which indicates that GnRHa can increase the number of adipocytes to a certain extent.

### Activation of GnRHR promotes the accumulation of lipid droplet

To elucidate the effect of the GnRH receptor on the production of lipid droplets during the process of adipocyte maturation, we detected the production of lipid droplets by oil-O staining. After the induction for 18 days, the lipid droplets were significantly increased following a dose-dependent treatment of diphereline (Figure 3B). Additionally, the appearance of lipid droplets occurred 1 to 2 days earlier compared with the control group, thus suggesting that activation of GnRH receptor can promote the accumulation of lipid droplets during the adipocyte maturation.

### Stimulating GnRHR inhibits the activation of the AMPK pathway

**Figure 4:**
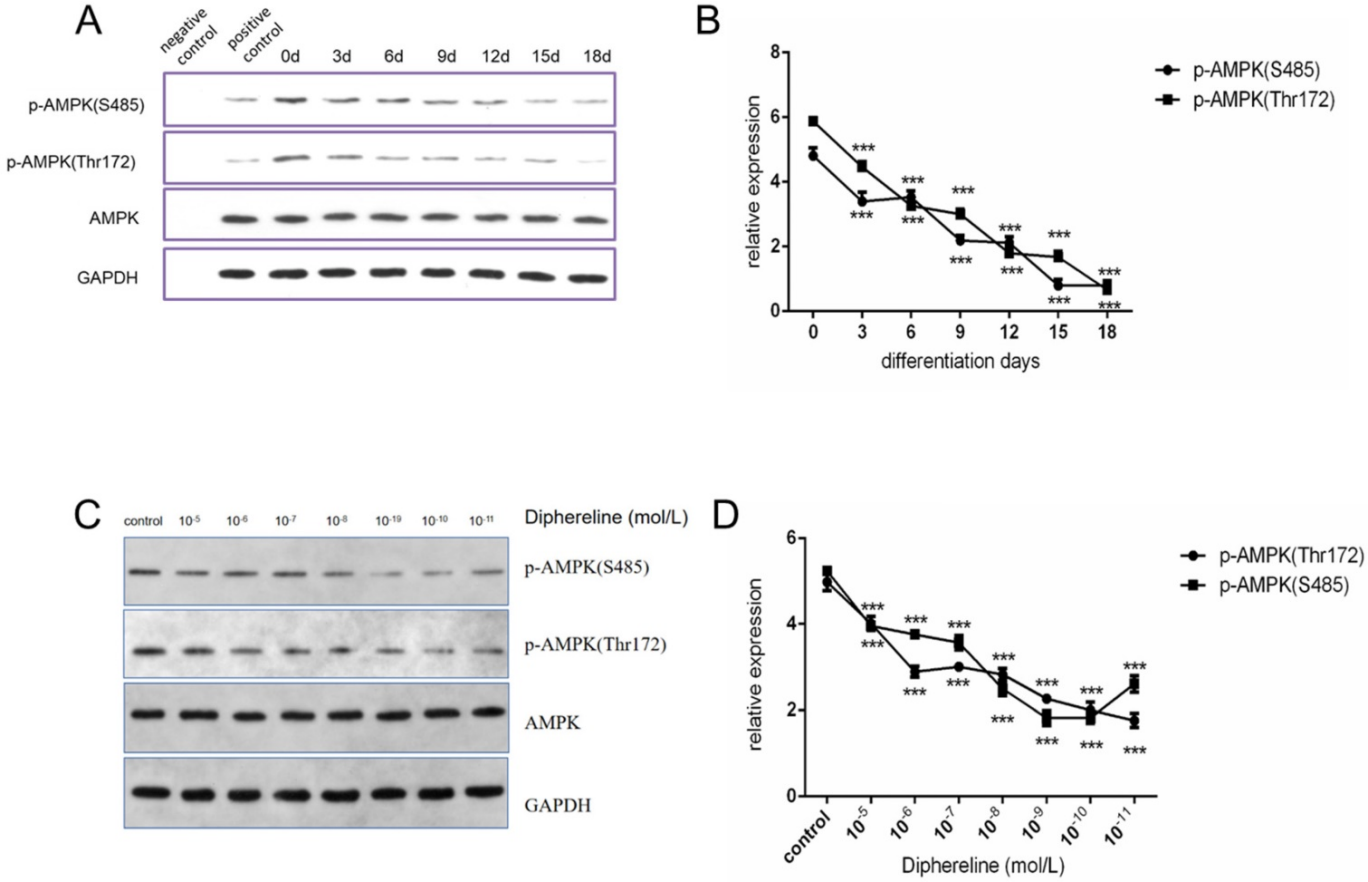
** Effect of culture time and GnRHa on p-AMPK (S485) and p-AMPK (Thr172). (A and B)** Western blot analysis of p-AMPK (Ser485), p-AMPK (Thr172), and AMPK during induction of HPA-s into adipocytes. **(C and D)** Western blot analysis of p-AMPK (Ser485), p-AMPK (Thr172), and AMPK using different concentrations of diphereline (***P<0.001).

The AMPK pathway has an important role in cellular energy metabolism, and it was reported that activation of the AMPK pathway could inhibit fat synthesis and promote lipolysis 25. In the present study, we found that phosphorylated AMPK was decreased during adipocyte maturation (Figure 4A and 4B), which was consistent with published papers. When treated with diphereline in different doses, the mature adipocytes showed the lower expression of phosphorylated AMPK (Figure 4C and 4D). However, the inhibition of AMPK phosphorylation was reversed when the concentration of diphereline was more than 10^-8^ mol/L, which was consistent with the pharmacological mechanism of the GnRHa drug. As previously reported, after the initial flare-up effect, GnRHa can decrease the stimulatory effect by decreasing the number and the sensitivity of GnRH receptor. Therefore, these data indicated that diphereline could inhibit the activation of AMPK during the induction of adipocyte maturation.

## Discussion

Obesity has been associated with many potentially serious health problems, including diabetes mellitus and cardiovascular diseases. In perimenopausal or menopausal women, patients with central precocious puberty, polycystic ovary syndrome, or a long-term GnRHa treatment, the high risk of obesity is correlated with high GnRH levels. Nevertheless, few studies addressed whether GnRH affects body weight.

The function of the GnRH hormone is to activate its receptor on the cell membrane and cytoplasm. GnRH receptor are mainly expressed in pituitary cells, as well as in other organs, such as ovaries, myometrium, endometrium, breast, prostate, placenta, and sex hormone-reactive tumors, including ovarian, endometrial, breast, and prostate cancers. However, it remains unclear whether GnRH receptor are expressed in adipose tissue. In our study, we first discovered the high expression of GnRH receptor in adipocytes, which indicated that GnRH might directly promote the occurrence and development of obesity through its receptor on adipocytes rather than central endocrine effects.

Adipogenesis is the process of maturation of adipocytes from preadipocytes accompanied by the accumulation of lipid droplets. In our study, we induced human preadipocytes into mature adipocytes. With the maturation of adipocytes and accumulation of lipid droplets, the expression of GnRH receptor gradually increased, which indicated that the expression of GnRH receptor was positively correlated with the maturity of adipocytes and accumulation of lipid droplets. These data suggest that GnRH receptor might promote adipocyte maturation and fat accumulation.

We found that activation of GnRH receptor by diphereline stimulated the proliferation of HPA-s, promoted adipocyte maturation, and increased the formation of lipid droplets in mature adipocytes, indicating that GnRH receptor can mediate the number of preadipocytes and the volume of adipocytes, thus promoting the occurrence of obesity.

Lipid metabolism is critical for the accumulation of lipid droplets in adipocytes during the development of obesity 26. Previous studies showed that many signal pathways are involved in lipid metabolism 27. Activation of the AMPK signal pathway mainly promotes lipolysis and inhibits lipid synthesis 28. In our study, we confirmed that the AMPK pathway is inhibited in the induction process of adipocytes.

Additionally, we also discovered that activation of the GnRH receptor by diphereline could inhibit the activity of AMPK pathway, aggravate the accumulation of lipid droplets, thus promoting obesity. This could explain the increased accumulation of lipid droplets during the induction process after being treated with diphereline. In summary, these findings suggest that the AMPK pathway is critical for the regulation of GnRH receptor on adipocytes.

The lower concentration of diphereline can obviously stimulate the proliferation of precursor cells and inhibit the phosphorylation of AMPK, but the effect is weakened at higher concentrations, which is exactly consistent with the pharmacological effects of GnRHa, first stimulated and then inhibited. In the future, animal model studies are needed to verify the relationship between GnRH and obesity. Moreover, it is necessary to further clarify the key molecules involved in GnRH promoting fat storage in adipocytes.

## Conclusion

The present study identified the high expression of GnRH receptor in adipocytes. Our results revealed that activation of GnRH receptor could increase the cell number of preadipocytes and the accumulation of lipid droplets. Mechanically, our data demonstrated that activation of GnRH receptor by diphereline could inhibit the AMPK pathway, which can aggravate the accumulation of lipid droplets. These findings provide a reasonable explanation for the increased body weight in perimenopausal or menopausal women, patients with central precocious puberty, polycystic ovary syndrome, or a long-term GnRHa treatment. Additionally, considering the effect of GnRH agonists on adipocytes, our study provides a useful reference to the clinical application of GnRH agonists in obese patients. Most importantly, this is the first study that investigated the relationship between GnRH and obesity, providing useful insight into how GnRH contributes to the development of obesity. These results enhance our understanding of the functional diversity of GnRH.

## Supplementary Material

Supplementary material.

## Abbreviations

GnRH: gonadotropin-releasing hormone
GnRHR: gonadotropin-releasing hormone receptor
GnRHa: GnRH agonist
BMI: body mass index
LH: luteinizing hormone
FSH: follicle-stimulating hormone
AMPK: AMP-activated protein kinases
HPA-s: human preadipocytes-subcutaneous
