# Supplementary figures and images for: GnRH receptor mediates lipid storage in female adipocytes via AMPK pathway
Source: Int J Med Sci. 2022 Aug 15;19(9):1442–50. doi: 10.7150/ijms.74335 (PMC9413554; doi:10.7150/ijms.74335)

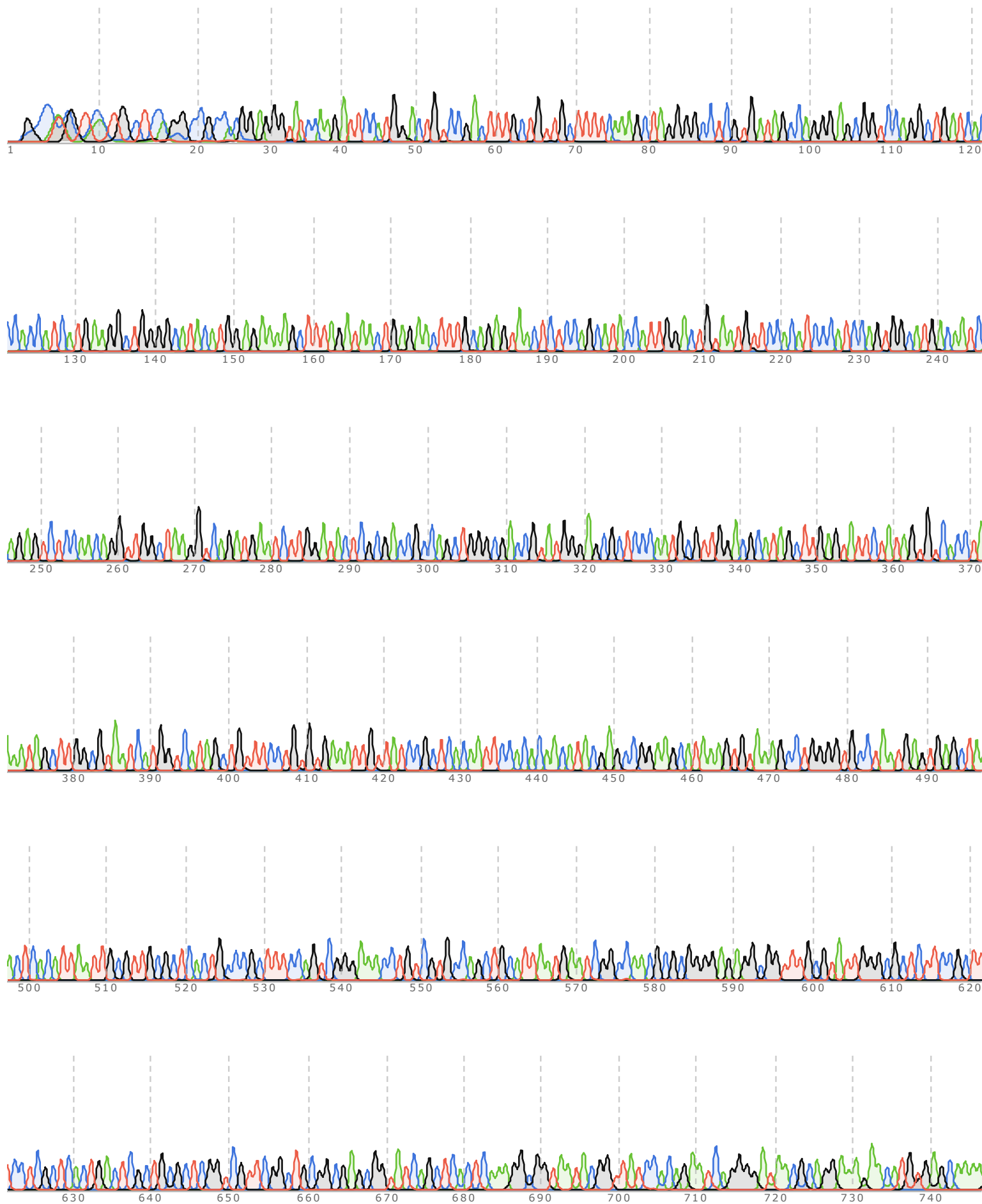

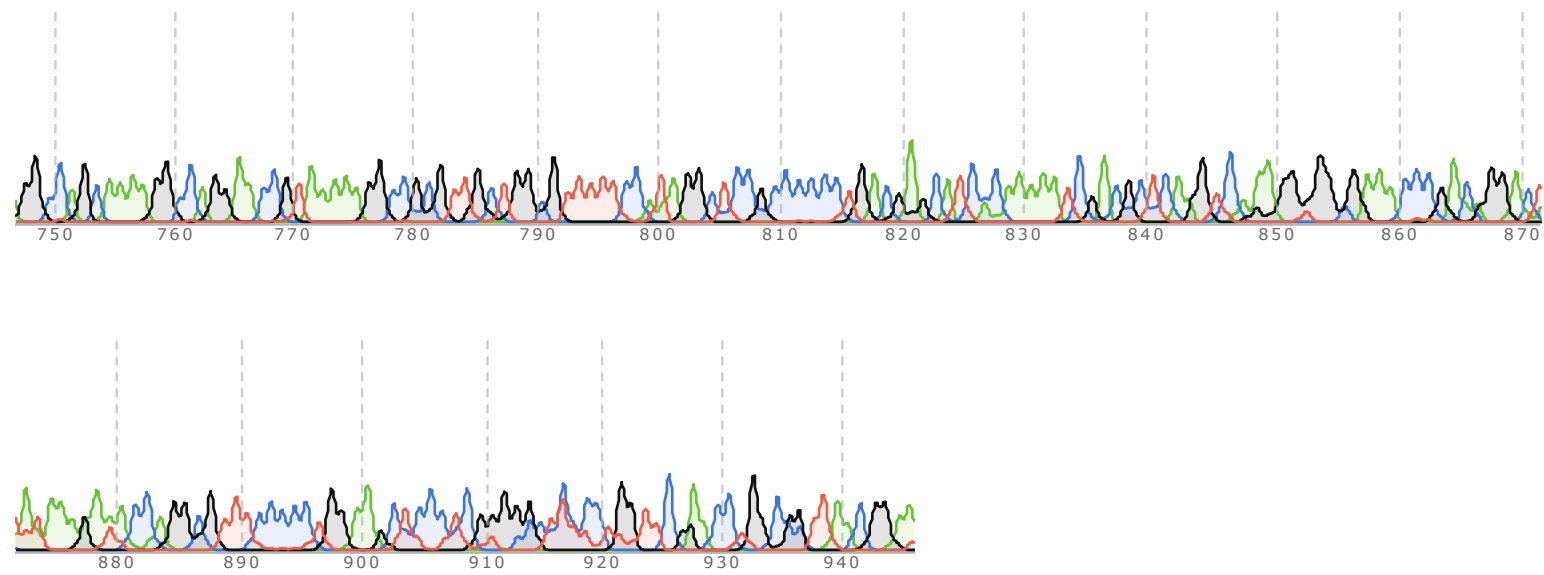

Supplement: Supplementary file 1 — Supplementary material. [file ijmsv19p1442s1.pdf]
